# Supplementary figures and images for: Stem cell-derived porcine macrophages as a new platform for studying host-pathogen interactions
Source: BMC Biol. 2022 Jan 14;20:14. doi: 10.1186/s12915-021-01217-8 (PMC8759257; doi:10.1186/s12915-021-01217-8)

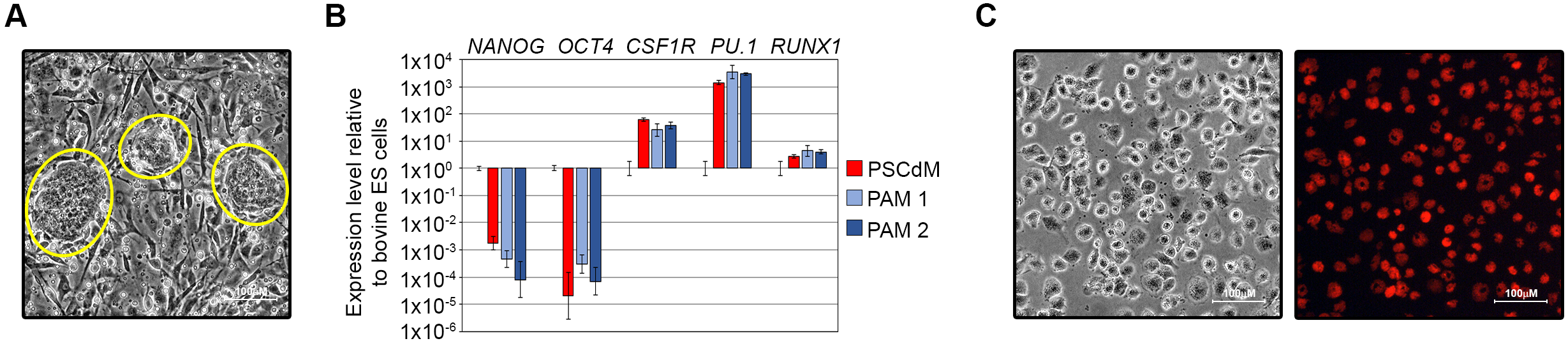

Supplement: Supplementary file 1 — Additional File 1: Fig. S1. Characterisation of bovine PSCdMs. (A) Bright-field image of bovine PSCs grown on mitotically-inactivated MEFs. Bovine PSC colonies are circled in yellow. (B) RT-qPCR analysis comparing expression of pluripotency markers (NANOG and OCT4) and macrophage markers (CSR1R, PU.1 and RUNX1) in primary bovine PAMs 62 and bovine PSCdMs relative to bovine PSCs. Mean and SD of three technical replicates. (C) Bright-field and fluorescent images of bovine PSCdMs containing phagocytosed pHrodo beads 22 h after exposure. [file 12915_2021_1217_MOESM1_ESM.tif]

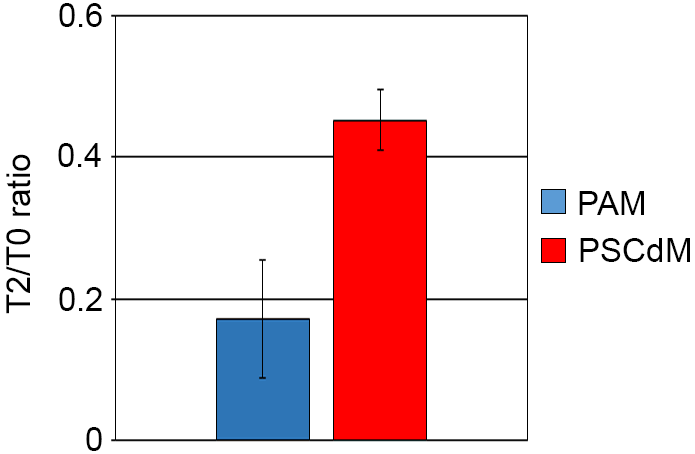

Supplement: Supplementary file 2 — Additional File 2: Fig. S2. Infection and clearance of Eschericia coli by porcine PSCdMs. Ratio of colony-forming Eschericia coli recovered from infected primary PAMs and porcine PSCdMs at 2 h post-infection relative to T0. Mean and SD of duplicate plates from two experiments. [file 12915_2021_1217_MOESM2_ESM.tif]

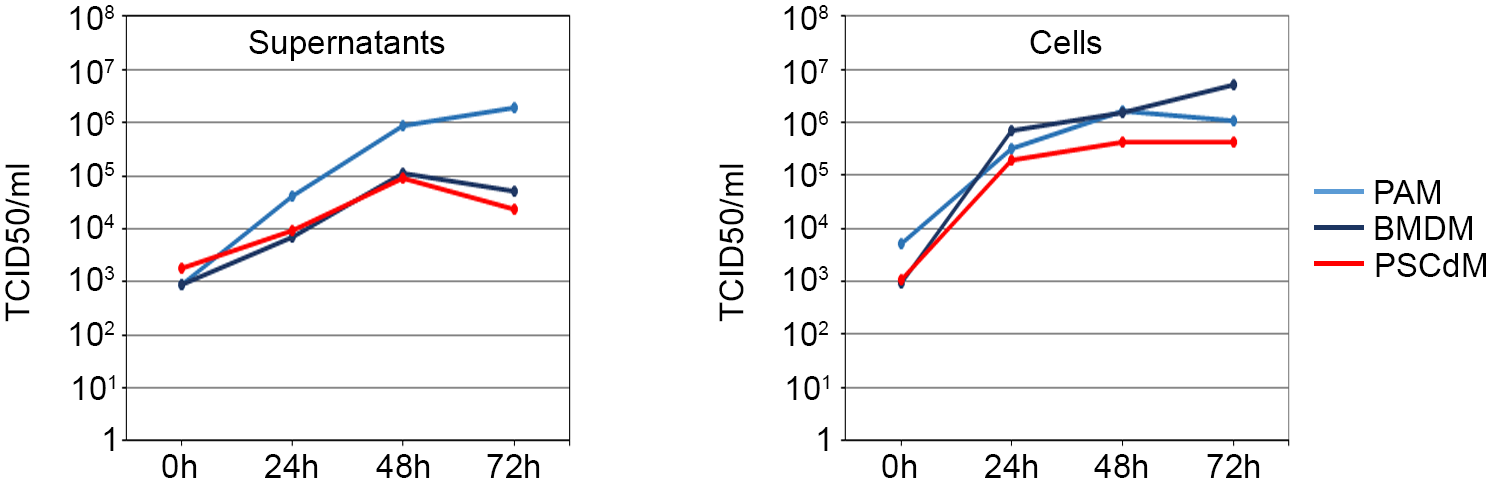

Supplement: Supplementary file 3 — Additional File 3: Fig. S3. Infection of porcine PSCdMs by ASFV as determined by a TCID50 assay. PAMs, BMDMs and PSCdMs were infected with ASFV (Benin 97/1 strain). Viral replication was determined by harvesting both supernatants and cells at 0, 24, 48 and 72 hpi, and titrating on pig BMDMs. TCID50 was calculated by the Spearman-Karber method. Data points represent mean of experimental duplicates. [file 12915_2021_1217_MOESM3_ESM.tif]

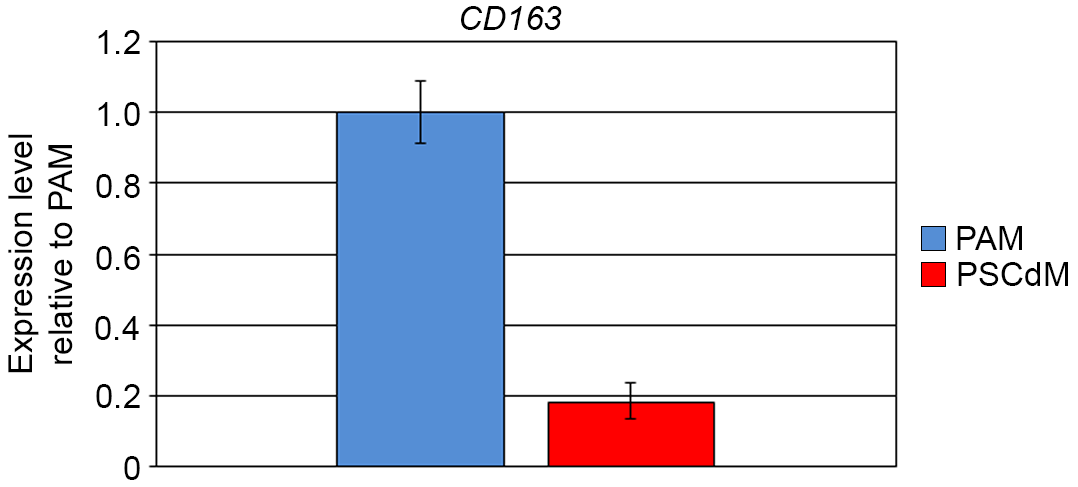

Supplement: Supplementary file 4 — Additional File 4: Fig. S4. CD163 expression levels in porcine PSCdMs and primary PAMs. RT-qPCR analysis comparing CD163 expression in primary PAMs and porcine PSCdMs. Mean and SD of duplicate samples from two experiments. [file 12915_2021_1217_MOESM4_ESM.tif]

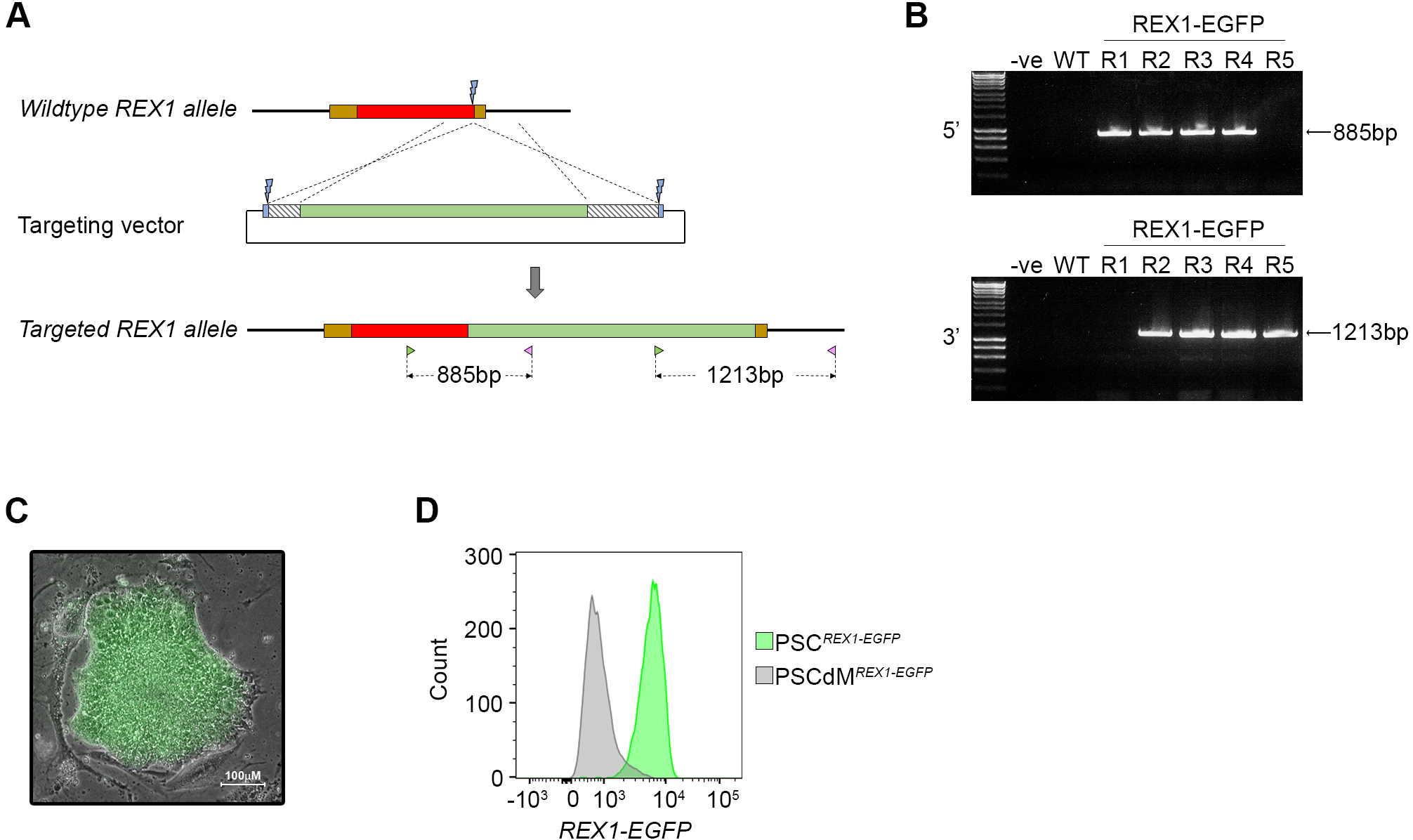

Supplement: Supplementary file 5 — Additional File 5: Fig. S5. Generation of Rex1-EGFP knock-in porcine PSCs. (A) Targeting diagram showing wild-type (top) and targeted (bottom) pig REX1 alleles generated using the PITCh targeting vector (middle) following CRISPR/Cas9-mediated homology-directed repair as indicated by the dotted lines. The targeting vector consisted of a T2A-EGFP-IRES-PURO-bGHpA cassette (green box) flanked by a 243 bp 5’ homology arm and a 534 bp 3’ homology arm (grey hashed boxes). The homology arms were flanked by inverted CRISPR/Cas9 guide sequences (blue boxes) that matched the endogenous CRIPSR/Cas9 cut site sequence (blue lightning bolts). Following co-electroporation of the targeting vector and Cas9/sgRNA RNP, puro-resistant PSC colonies were generated in which the REX1 stop codon had been replaced with the reporter/selection cassette at the 3’ end of the REX1 coding exon (red box) immediately upstream of the 3’ UTR (brown box). Non-coding genomic sequence and plasmid backbone sequence are represented by thick and thin black lines respectively, and 5’ and 3’ UTRs by brown boxes. Confirmation of correctly targeted clones was performed at both the 5’ and 3’ end of the integration site using forward and reverse primers flanking the 5’ and 3’ homology arms respectively. Expected PCR product sizes are indicated. (B) Five puro-resistant, EGFP+ clones were genotyped by PCR using the primers indicated in panel A. Clones R2, R3 & R4 showed the expected products at both the 5’ and 3’ ends of the integration site. Water and wild-type, parental porcine PSC genomic DNA were used as negative controls (-ve and WT respectively). (C) Compound bright-field and fluorescent image of a REX1-EGFP positive porcine PSC colony. (D) Flow cytometry analysis of porcine REX1-EGFP PSCs and PSCdMs. [file 12915_2021_1217_MOESM5_ESM.tif]

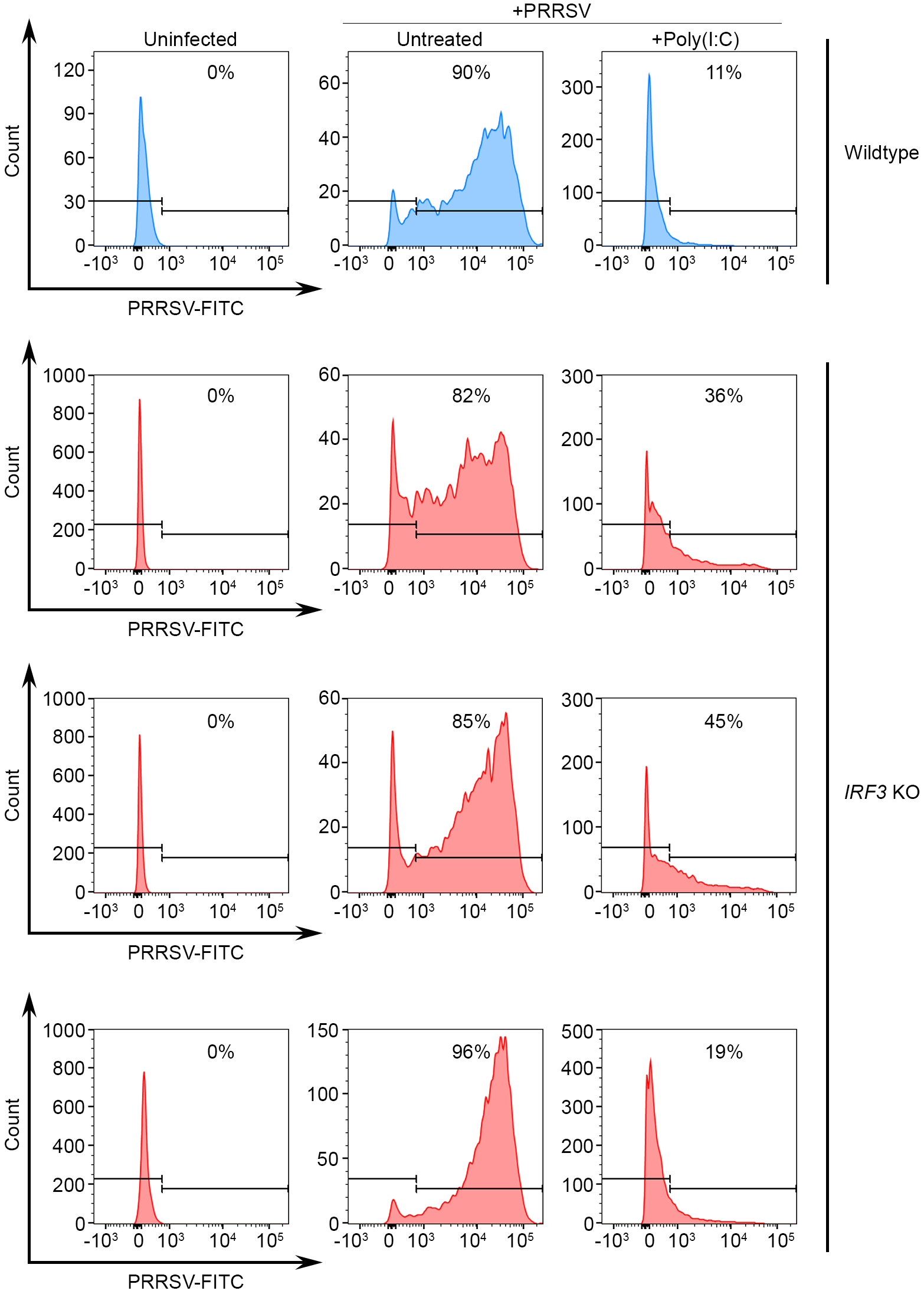

Supplement: Supplementary file 6 — Additional File 6: Fig. S6. Infection of IRF3 KO porcine PSCdMs with PRRSV. Flow cytometry analysis for PRRSV nucleocapsid protein in three IRF3 knock-out (KO) porcine PSCdMs clones relative to the wild-type parental line. Plots represent uninfected (left), untreated/infected (middle) and poly(I:C)-treated/infected (right). For poly(I:C) treatment cells were pre-treated with 25 μg/ml for 3 h prior to infection. [file 12915_2021_1217_MOESM6_ESM.tif]

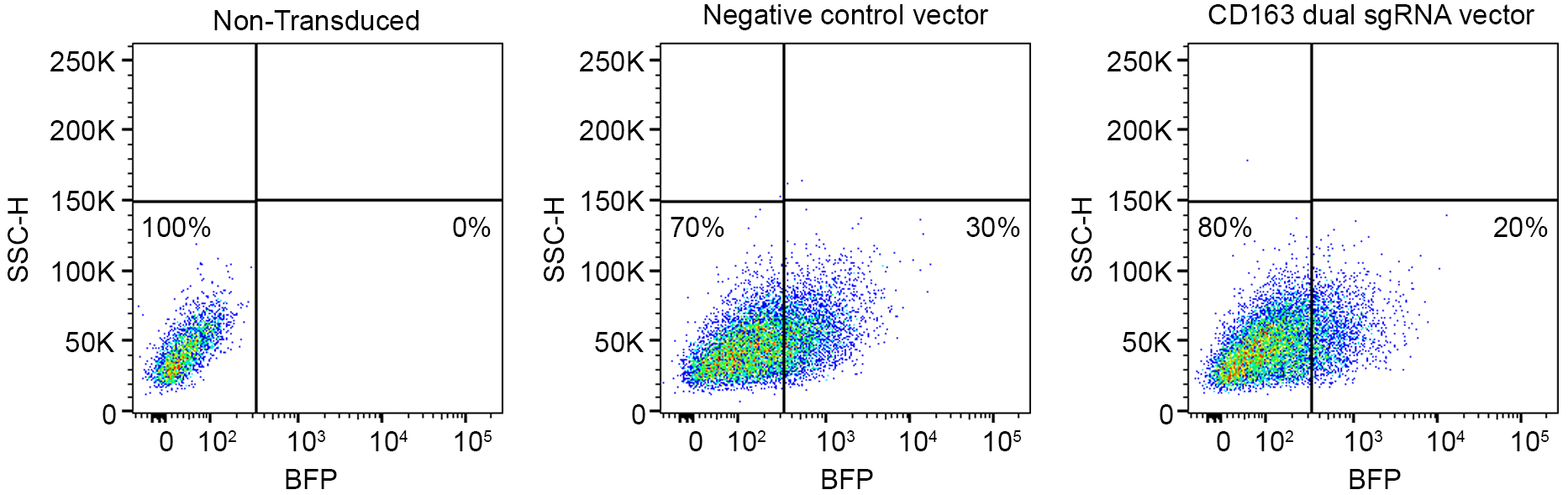

Supplement: Supplementary file 7 — Additional File 7: Fig. S7. Lentiviral transduction of porcine PSCdMs with a CD163 dual guide lentivirus. Flow cytometry data for porcine PSCdMs transduced with a lentiviral dual-expression vector expressing the CD163 CRISPR guide RNAs SL26 and SL68 55 (right panel) or a negative control vector containing no guide sequences (middle panel) relative to non-transduced cells (left panel). BFP+ve cells were sorted seven days post-transduction using the conservative FACS gate shown. [file 12915_2021_1217_MOESM7_ESM.tif]
